# Acute Surge of Atypical Memory and Plasma B-Cell Subsets Driven by an Extrafollicular Response in Severe COVID-19

**DOI:** 10.3389/fcimb.2022.909218

**Published:** 2022-07-08

**Authors:** Taeseob Lee, Yuri Kim, Hyun Je Kim, Na-Young Ha, Siyoung Lee, BumSik Chin, Nam-Hyuk Cho

**Affiliations:** ^1^ Department of Digital Health, Samsung Advanced Institute for Health Sciences & Technology, Sungkyunkwan University, Seoul, South Korea; ^2^ Discovery department, Biomarker Laboratory, Geninus Inc., Seoul, South Korea; ^3^ Institute of Endemic Diseases, Medical Research Center, Seoul National University, Seoul, South Korea; ^4^ College of Medicine, Genome Medicine Institute, Seoul National University, Seoul, South Korea; ^5^ School of Medicine, Biomedical Research Institute, Chungnam National University, Daejeon, South Korea; ^6^ Department of Internal Medicine, National Medical Center, Seoul, South Korea; ^7^ Department of Microbiology and Immunology, College of Medicine, Seoul National University, Seoul, South Korea; ^8^ Department of Biomedical Sciences, College of Medicine, Seoul National University, Seoul, South Korea; ^9^ Bundang Hospital, Seoul National University, Seongnam, South Korea; ^10^ Wide River Institute of Immunology, Seoul National University, Hongcheon, South Korea

**Keywords:** B cells, extrafollicular response, COVID-19, antibody response, plasma cell

## Abstract

**Background:**

Despite the use of vaccines and therapeutics against the coronavirus disease 2019 (COVID-19) pandemic, this severe disease has been a critical burden on public health, whereas the pathogenic mechanism remains elusive. Recently, accumulating evidence underscores the potential role of the aberrant B-cell response and humoral immunity in disease progression, especially in high-risk groups.

**Methods:**

Using single-cell RNA (scRNA) sequencing analysis, we investigated transcriptional features of B-cell population in peripheral blood from COVID-19 patients and compared them, according to clinical severity and disease course, against a public B-cell dataset.

**Results:**

We confirmed that acute B cells differentiate into plasma cells, particularly in severe patients, potentially through enhanced extrafollicular (EF) differentiation. In severe groups, the elevated plasma B-cell response displayed increased B-cell receptor (BCR) diversity, as well as higher levels of anti–severe acute respiratory syndrome coronavirus 2 (anti–SARS-CoV-2) spike antibodies in plasma, than those in moderate cases, suggesting more robust and heterogeneous plasma cell response in severe COVID-19 patients. Trajectory analysis identified a differentiation pathway for the EF B-cell response from active naïve to atypical memory B cells (AM2), in addition to the emergence of an aberrant plasma cell subset (PC2), which was associated with COVID-19 progression and severity. The AM2 and PC2 subsets surged in the acute phase of the severe disease and presented multiple inflammatory features, including higher cytokine expression and humoral effector function, respectively. These features differ from other B-cell subsets, suggesting a pathogenic potential for disease progression.

**Conclusion:**

The acute surge of AM2 and PC2 subsets with lower somatic hypermutation and higher inflammatory features may be driven by the EF B-cell response during the acute phase of severe COVID-19 and may represent one of the critical drivers in disease severity.

## Introduction

The coronavirus disease 2019 (COVID-19) pandemic resulted from the emergence of a life-threatening disease caused by the virus severe acute respiratory syndrome coronavirus 2 (SARS-CoV-2). Currently, there have been over 450 million confirmed cases of COVID-19, which have led to over 6 million deaths globally (https://covid19.who.int/). The clinical course of SARS-CoV-2 infection is highly variable, ranging from often asymptomatic infection in healthy young adults to severe pneumonia and multisystem failure, which is more prevalent in the elderly and those with comorbidities. The immunological response has been implicated both in overcoming infection alongside contributing to the severe disease (Garcia, 2020).

Recent studies demonstrate that the germinal center (GC) response, which is important for long-term immunity, is disrupted in severe COVID-19 (Kaneko et al., 2020; Woodruff et al., 2020). Furthermore, there is evidence of broad changes from COVID-19 in B-cell populations, such as an expansion of B-cell clones containing somatic hypermutations (SMHs) (Kreer et al., 2020; Nielsen et al., 2020). Normal effector B- and T-cell responses control acute viral infections and provide the foundation for the subsequent development of specific immunological memory. In contrast to extensive T-cell studies, the potential role of human effector B-cell responses during the pathogenic condition remains poorly understood beyond the expansion of antibody-secreting cells (ASCs). Indeed, in mouse studies, the early primary antiviral responses are mediated by extrafollicular (EF) differentiation of naïve B cells into short-lived ASCs independent of traditional GC incorporation (Lam et al., 2020). However, while EF responses in different models are heterogeneous in their requirement for T-cell assistance, it is now established that the mouse EF B-cell responses can undergo affinity maturation and generate both memory and long-lived plasma cells, even under T-cell–independent conditions (Di Niro et al., 2015; Allman et al., 2019). Moreover, previous studies into human EF B-cell responses have been hindered by a lack of proper phenotypic identification of their cellular components. Comparatively, recent studies on severe COVID-19 patients presented evidence, which suggests that EF B-cell activation strongly correlates with the expansion of ASC (plasma cells), alongside the early production of high concentrations of SARS-CoV-2–specific neutralizing antibodies (Woodruff et al., 2020). Nevertheless, these patients also presented elevated inflammatory biomarkers, multiple organ failure, and, in some cases, death. Ultimately, these outcomes suggest a pathogenic role of the enhanced EF B-cell response in COVID-19 progression, a role similar to that previously demonstrated in autoimmune disorders, such as human systemic lupus erythematosus (SLE) (William et al., 2002; Tipton et al., 2015; Woodruff et al., 2020). Indeed, the frequency of CD19^+^ B cells increased in severe COVID-19 cases compared to mild cases. Moreover, transitional (TR) B-cell subsets increased in mild and moderate cases, whereas the memory B compartment decreased in severe and critical cases, while the ASCs increased in accordance with the disease severity (Sosa-Hernandez et al., 2020). Similarly, there have been significant increments in the double-negative (DN) CD27^−^ IgD^−^ B-cell subsets (DN2 and DN3), alongside a relevant decrease in the DN1 B-cell subpopulation in conjunction with COVID-19 case severity and the patient’s outcome (Cervantes-Diaz et al., 2022). These DN cell numbers also appeared to correlate with pro- or anti-inflammatory signatures, respectively (Cervantes-Diaz et al., 2022). These pathogenic changes in the B-cell compartment previously observed in SLE, hepatitis C, and HIV infection (Holla et al., 2021; Sanz et al., 2019) have now similarly been observed COVID-19 (Sosa-Hernandez et al., 2020). The DN2 cells derive from naïve cells poised to generate plasmablasts through an EF pathway (Jenks et al., 2018). However, the underlying mechanisms and pathogenic phenotypes of this aberrant EF B-cell response are yet to be fully defined for COVID-19.

Therefore, to investigate the mechanistic basis of the potential pathogenic contribution of B-cell subsets in COVID-19 progression, we enriched plasma B-cell populations in peripheral blood samples collected from nine COVID-19 patients presenting with either moderate or severe disease symptoms during the convalescent phase. The transcriptional features of B-cell subtypes were subsequently analyzed by single-cell RNA (scRNA) sequencing and found to correlate with disease severity. In addition, our dataset was validated by transcriptional markers of B cells and combined with the public B-cell scRNA dataset, a previously collated COVID-19 cohort collected during the acute stage, in addition to, from healthy controls (Bernardes et al., 2020; 10X Genomics, 2022). This integrative analysis enabled us to characterize phenotypic features observed in various B-cell subsets and their dynamics according to the COVID-19 disease course and its severity.

## Materials and Methods

### COVID-19 Patients and Ethics Statement

COVID-19 patients were recruited at a referral care center in Seoul, Republic of Korea. COVID-19 was confirmed in eligible patients following a nasopharyngeal swab by a positive PCR test (KogeneBiotech, Seoul, South Korea) for SARS-CoV-2, whereas pneumonia was confirmed by a radiologic image. Patients nearing discharge following the recovery from COVID-19 were invited to participate in this study and whole-blood samples for cellular analyses were taken after their informed consent. The institutional ethics and research committees approved the subsequent protocol (H-2004-116-011) in compliance with the Helsinki declaration.

### Patients’ Data Collection

The demographic and clinical data of patients are summarized in **Table 1**. The severity grade is classified as the worst condition during the disease course and is graded according to the WHO Ordinal Scale for Clinical Improvement (World Health Organization, 2020) and NIH treatment guidelines (National Institute of Health, 2021). None of the patients were vaccinated.

**Table 1:** Clinical features of the coronavirus disease 2019 (COVID-19) patients.

Case ID.	Stage	Sex	Age	Comorbidities	WHO Scale (max)	NIH spectrum(max)	CRP(max, mg/dl)	Anti-RBD IgG titer (ELISA)	Symptom onsetto sample (day)	Data Source
A01	acute	male	75–79	hypertension, COPD	4	severe	10.1	–	8	(Bernardes et al., 2020)
A02	acute	male	55–59	none	8	critical	35.8	–	19	(Bernardes et al., 2020)
A04	acute	male	60–64	hypertension	4	severe	24.1	–	9	(Bernardes et al., 2020)
A05	acute	male	45–49	asthma, psoriasis, diabetes	4	severe	16.8	–	6	(Bernardes et al., 2020)
A06	acute	male	70–74	hypertension	4	severe	4.9	–	12	(Bernardes et al., 2020)
A07	acute	male	25–29	none	2	moderate	–	–	45	(Bernardes et al., 2020)
A08	acute	female	60–64	kidney transplant, hypertension	4	severe	21.7	–	18	(Bernardes et al., 2020)
A09	acute	male	55–59	hypercholesterolemia	4	severe	11.6	–	18	(Bernardes et al., 2020)
A10	acute	male	70–74	ESRD, hypertension, diabetes	4	severe	28.8	–	9	(Bernardes et al., 2020)
A11	acute	female	75–79	cerebrovascular accident	3	moderate	0.6	–	4	(Bernardes et al., 2020)
A12	acute	female	50–54	none	6	critical	34.8	–	29	(Bernardes et al., 2020)
C01	convalescent	male	76	hypertension	5	severe	115.3	31,506	78	This Study
C02	convalescent	male	60	hypertension	2	moderate	201.7	7,327	28	This Study
C03	convalescent	female	56	diabetes	5	severe	43.5	29,745	35	This Study
C05	convalescent	male	50	none	4	severe	68.8	34,291	40	This Study
C06	convalescent	male	36	diabetes	2	moderate	44.0	2,811	42	This Study
C08	convalescent	male	55	hypertension, diabetes	2	moderate	2.3	5,579	27	This Study
C09	convalescent	male	72	diabetes, ischemic heart disease	4	severe	<0.3	10,429	61	This Study
C10	convalescent	male	60	none	2	moderate	2.9	4,841	43	This Study
C11	convalescent	male	26	none	1	moderate	0.3	1,622	43	This Study

COPD, chronic obstructive pulmonary disease; ESRD, end-stage renal disease.

### Plasma Collection and PBMC Isolation

The collected blood samples were centrifuged at 1,000*g* for 10 min and the plasma was stored at −150°C. Precipitated blood cells were diluted with Dulbecco’s phosphate-buffered saline (DPBS; WelGENE Inc., Daegu, South Korea) and the peripheral blood mononuclear cells (PBMCs) were isolated by density gradient centrifugation using a Ficoll-Paque Plus (GE Healthcare, Uppsala, Sweden). The subsequent samples were cryopreserved in a CELLBANKER^®^ 1 (Nippon Zenyaku Kogyo, Fukushima, Japan) at −80°C prior to analysis.

### Enrichment of B Cells

Initially, we tried to enrich plasma B cells from PBMCs in order to focus on transcriptional feature of EF B-cell responses in COVID-19 patients. We used magnetic-activated cell sorting (MACS) using the Human Plasma Cell Isolation Kit II (Miltenyi Biotec, Bergisch Gladbach, Germany) according to the manufacturer’s instructions. Briefly, frozen PBMCs were thawed in RPMI 1640 (Gibco) containing 10% fetal bovine serum (FBS) and washed with MACS buffer (PBS pH 7.2; 0.5% bovine serum albumin; 2 mM EDTA). Following cell counting, the PBMCs were pooled to provide sufficient cell numbers for MACS and scRNA sequencing. Non-plasma cells are magnetically labeled with a cocktail of biotin-conjugated monoclonal antibodies (anti-CD2, CD3, CD10, CD13, CD14, CD15, CD22, CD34, CD56, CD123, and CD235a) and anti-biotin microbeads. The labeled cells are subsequently depleted by separation over a MACS Column. In the second step, the unlabeled cells in effluent were incubated with anti-CD38 microbeads and positively selected.

### ELISA

SARS-CoV-2 spike receptor-binding domain (RBD) protein (Sino Biological, Beijing, China) was incubated in 50 mM carbonate buffer (pH 9.6) and coated on an immunoplate (Thermo Fisher Scientific, Waltham, USA) at 4°C for 18 h. After blocking with 5% skim milk (Becton, Dickinson and Company, Franklin Lakes, USA), the plasma extracted from COVID-19 patients was serially diluted in a 5% skim milk solution and left to react with the viral antigen at room temperature for 1 h. Afterward, the secondary antibody, a goat anti-human immunoglobulin G (IgG) conjugated with horseradish peroxidase, was incubated at room temperature for 1 h, followed by a 3,3′,5,5′-tetramethylbenzidine substrate reaction. Absorbance was measured by a microplate reader (TECAN, Männedorf, Switzerland) at 450 nm. A cutoff value was established in accordance with previous studies (Frey et al., 1998) as “the average absorbance of plasma from four healthy volunteers plus 5.077 times the standard deviation (SD)”. The titer of SARS-CoV-2 RBD-specific IgG was calculated by non-linear regression analysis using the 4PL sigmoidal dose curve equation on Prism 9.1.2 (GraphPad Software, San Diego, USA).

### Virus Neutralization Assay

Plasma was heat-inactivated at 56°C for 30 min and serially diluted from 1:10 to 1:6,250 in media. Diluted plasma and equal volume of virus solution containing 50 foci-forming unit of SARS-CoV-2 were mixed and incubated at 4°C for 1 h. Then, the solutions were added to VeroE6 cells and cultured for 4 days under overlay medium including 0.8% methylcellulose (Sigma-Aldrich) and 2% FBS in Dulbecco’s modified Eagle’s medium (Gibco). Cells were fixed with 4% paraformaldehyde and permeabilized by 0.2% Trixon X-100 in PBS (Sigma-Aldrich). SARS-CoV-2–infected foci were immunostained with anti–SARS-CoV-2 N protein rabbit serum and goat anti-rabbit IgG antibody conjugated with alkaline phosphatase (Invitrogen). Stained foci were visualized by reacting with nitro blue tetrazolium/5-bromo-4-chloro-3-indolyl-phosphate (Roche, Basel, Switzerland). Fifty percent foci reduction neutralization titer (FRNT_50_) was calculated by non-linear regression analysis using the 4PL sigmoidal dose curve equation on Prism 9.1.2 (GraphPad Software).

### Single-Cell RNA Sequencing

Enriched CD38^+^ plasma B cells were washed with chilled DPBS and counted. Following, 20,000 live cells were loaded into a chromium chip well and scRNA sequencing was performed using the Chromium Single Cell 5′ Library & Gel Bead Kit v1 and V(D)J Reagent Kits (10X Genomics, Pleasanton, USA) according to the manufacturer’s instruction. The cDNA library quality was determined using an Agilent Bioanalyzer (Agilent Technologies, Santa Clara, USA) and next-generation sequencing was performed using Nextseq550 (Illumina, San Diego, USA). Single-cell data were demultiplexed into the individual patients by single-nucleotide polymorphism (SNP) analysis using isolated genomic DNA from PBMC samples.

### Single-Cell RNA Analysis

The gene expression matrix established from nine convalescent patients was generated by Cell Ranger V3.1 (Zheng et al., 2017) and then each cell was assigned to the nine patients by calling and matching SNPs using demuxlet (Kang et al., 2018) and sourporcell (Heaton et al., 2020) programs. Cells assigned to multiple patients were filtered from the dataset as were the cells presenting a high mitochondrial gene percentage (>30%). Seurat R package v4.0.1 (Hao et al., 2021) was utilized for normalization, variable gene selection, principal components analysis calculation, Uniform Manifold Approximation, and Projection (UMAP) analysis alongside defining differentially expressed gene (DEG). The criteria for defining DEGs are the following: percentage of cells in the cluster expressing target gene >10%, log_2_ fold change >0.25, and an adjusted *p*-value < 0.01. The B-cell dataset from convalescent patients was integrated with the COVID-19 dataset for the acute phase (11 patients) and healthy (9 controls) PBMC datasets available in public databases (Bernardes et al., 2020; 10X Genomics, 2022) using 10X Genomics and the R package harmony (Korsunsky et al., 2019). During integration with datasets from the public databases, we extensively validated the presence of B-cell–specific transcripts (CD19, CD22, CD 27, or CD38) for selection of pure B-cell population and removed incompatible cells and other contaminated cellular populations, such as myeloid cells (CD14^+^), T cells (CD3D^+^), natural killer (NK) cells (NCR1^+^), and platelets (PPBP^+^), from the datasets (**Supplementary Figure 1**). Although we applied enriched plasma B cells in PBMCs from convalescent patients, our dataset included decent levels of naïve and memory B cells, in addition to plasma cells and plasmablasts (**Supplementary Figure 2**). The combined B-cell dataset (16,920 cells) was annotated into major subtypes using DEGs as previously reported (Sanz et al., 2019; Woodruff et al., 2020). To assess the potential association of EF B-cell subsets with disease fatality, we analyzed the scRNA dataset for the B-cell population collected from another cohort of COVID-19 patients, which included data pertaining to patients who both survived and deceased following admittance to intensive care units (ICU) (Bost et al., 2021). The SMH rate of immunoglobulin transcripts was calculated by IgBlast v1.17.1 (Ye et al., 2013), then B-cell subsets were further annotated using both DEGs and the average value of SMH rate data. Trajectory analysis was conducted by monocle3 R package v1.0.0 (Trapnell et al., 2014), and the contour trajectory UMAP was visualized by geom_density_2d function from ggplot2. ROGUE v1.0 was used to calculate the expression purity of each patient group: acute moderate, acute severe, convalescent moderate, convalescent severe, or healthy control (Liu et al., 2020). To ensure unbiased analysis, we inspected cellular distribution of each patient on the trajectory result (**Supplementary Figures 3** and **4**). To define any unique transcriptional signatures of EF B cells, we analyzed the DEGs for EF B cells in comparison with those from other B-cell subsets. The significant DEGs were represented as a volcano plot. Following, functional enrichment analysis was performed by gprofiler2 v0.2.0 to search related enriched Gene Ontology (GO) terms (Reimand et al., 2007). R package escape v1.0.0 was also utilized to calculate potential detailed enrichment scores.

### Single-Cell B-cell Receptor Analysis

B-cell receptor (BCR) sequence data were aligned and annotated by Cell Ranger V3.1 (Zheng et al., 2017), and the cellular index was matched to the gene expression data by Seurat R package v4.0.1 (Hao et al., 2021). Each B-cell immunoglobulin heavy chain (IgH) isotype was identified with a V(D)J annotation. The homogeneity scores for B-cell response sequences were calculated by distance among the CDRH3 region of the IgH sequences using distToNearest function in shazam v1.0.2 (Gupta et al., 2015). Linear regression was conducted by lm function in R and confidence interval for the regression was displayed by geom_smooth function of ggplot2 package v3.3.5. Correlation of anti-spike RBD titer with average values of homogeneity score calculated in each patient was also assessed.

## Results

### Enhanced Plasma Cell Responses in Severe COVID-19

**Figure 1:**
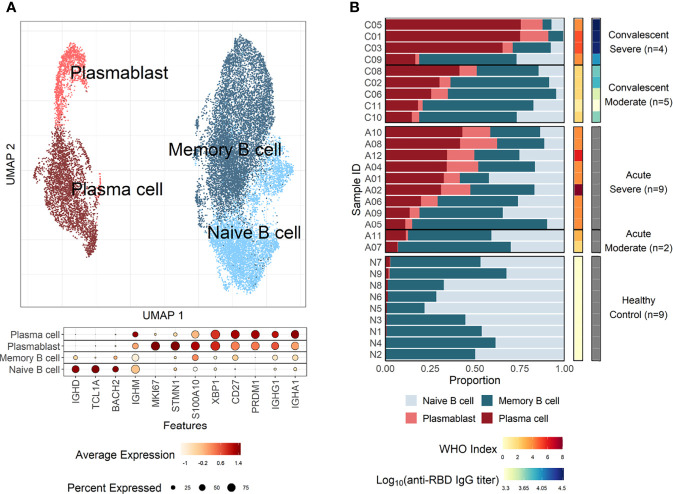
B-cell compositions and profiles from COVID-19 patients and healthy individuals. **(A)** UMAP analysis with four clusters annotated by distinct markers of B-cell subtypes. Differentially expressed genes (DEGs) of each cluster are stated in a dot plot with diameter from the percentage of cells expressing each given gene, and with color for expression levels. **(B)** Proportion bar plot of four B-cell subtypes for each patient categorized by disease course and severity (WHO index). Log-scaled anti–receptor-binding domain (RBD) immunoglobulin G (IgG) titer is presented as colored code on the right-hand side.

Enriched plasma B cells from nine patients in convalescent phase were analyzed using scRNA sequencing to characterize the B-cell responses and their molecular activity. The B-cell subsets transcriptome data were also integrated with B-cell scRNA sequencing datasets from 11 patients in the acute phase of COVID-19 and 9 healthy controls, as previously reported (Bernardes et al., 2020; 10X Genomics, 2022). Since intensive validation of B-cell scRNA datasets from current study and public databases (Bernardes et al., 2020; 10X Genomics, 2022) using integrated UMAP analysis revealed significant contamination of myeloid cells, T cells, NK cells, and platelets (**Supplementary Figure 1**), we removed them and used only pure B-cell population for further analysis. These integrated B-cell subsets were initially classified into four groups according to cellular signatures (Sanz et al., 2019), including IGD, CD27, and IGHA: naïve B cells, memory B cells, plasmablasts, and plasma cells (**Figure 1A**). Although we used enriched CD38^+^ plasma B cells for scRNA analysis, we detected decent level of naïve (299 cells, 17.8%) and memory (827 cells, 49.3%) B cells, in addition to plasma cells (462 cells, 27.6%) and plasmablasts (88 cells, 5.3%) in our dataset (**Supplementary Figures 1** and **2**). This might be due to inefficiency of plasma cell isolation kit. As reported previously (Sosa-Hernandez et al., 2020; Bernardes et al., 2020), a higher proportion of plasma cells (average: 57.43%) was detected among B cells in severe COVID-19 patients compared to moderate cases during the convalescent stage (average: 25.87%) (**Figure 1B**), although we applied the same procedures for enrichment of plasma B cells in the convalescent specimens. This pattern was reproducibly observed in the B-cell population from COVID-19 patients in the acute phase (severe: 27.93%, moderate: 9.10%). In contrast, the plasma cells were barely detected in healthy controls (average: 1.05%). The proportion of plasma cells among PBMCs was also significantly higher in acute phase of COVID-19 patients than in healthy control group (**Supplementary Figure 5**). Moreover, patients demonstrating moderate symptoms presented more memory B cells than severe patients, both in acute (severe: 36.39%, moderate: 53.98%) and convalescent phases (severe: 21.79%, moderate: 53.23%). In addition, plasmablasts were on average higher in COVID-19 patients than in the healthy control group, where naïve B cells (average: 54.49%) formed the primary population. IgG antibody titers against the SARS-CoV-2 spike RBD in plasma from the convalescent patients were also significantly higher in the severe group than in moderate cases. These results indicate that the active commitment of B-cell differentiation into plasma cells is one of the hallmark responses observed in COVID-19, which correlates with findings of previous studies (Sosa-Hernandez et al., 2020; Bernardes et al., 2020).

### Increased B-cell response Heterogeneity but Reduced Repertoire Specific to SARS-CoV-2 in B Cells From Severe COVID-19

**Figure 2:**
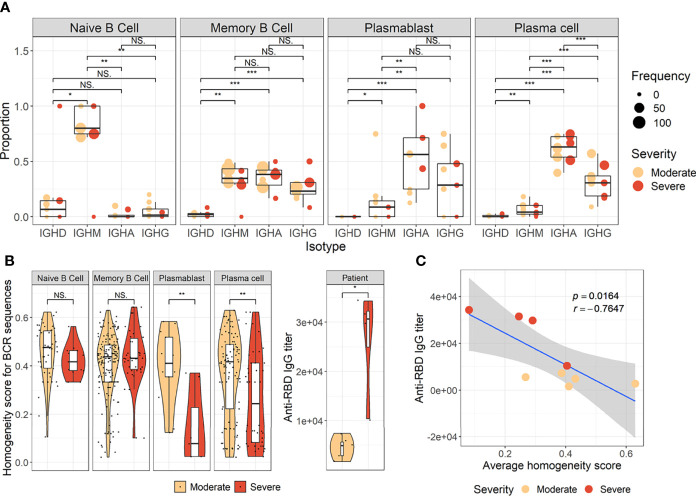
Characteristics of B-cell response transcripts in coronavirus disease 2019 (COVID-19) patients according to cellular subtypes and disease severity. **(A)** Boxplots of the indicated IgH isotype proportion. Each dot represents the proportion of each indicated isotype from each patient and the circle size displays the number of cells identified. **(B)** Violin plots presenting BCR homogeneous scores (left panels) and anti–severe acute respiratory syndrome coronavirus 2 (anti–SARS-CoV-2) spike RBD-specific IgG titers (right panel). Statistical significance was calculated by Wilcoxon rank sum test: ***p < 0.0001; **p < 0.001; *p < 0.01. NS, not significant. **(C)** Correlation of anti-RBD-specific IgG titers with average homogeneity score of COVID-19 patients. Statistical significance was calculated by Pearson’s correlation.

The pathway and characteristics of B-cell differentiation in COVID-19 were further investigated through analysis of single-cell B-cell response repertoire sequences in B cells obtained from convalescent patients. Distinct immunoglobulin isotypes were dominated when the proportion of each isotype sequence was assessed in the four B-cell subtypes (**Figure 2A**). Notably, IGHA isotypes were significantly more enriched in plasma cells and plasmablasts. Naïve B cells primarily encoded IGHM, whereas memory B cells showed a relatively equal proportion of switched isotype responses with IGHA, IGHG, and IGHM (**Figure 2A**). This preferential dominance of specific isotypes among the different B-cell subsets was consistently observed regardless of COVID-19 severity. Especially, the presence of enriched IGHA transcripts in plasmablasts and plasma cells from COVID-19 patients clearly indicates that active differentiation and proliferation of B cells with IgA isotype class-switching are dominant during the pulmonary infection. These findings are consistent with a prevalence of IgA responses in COVID-19 patients (Galson et al., 2020; Zeng et al., 2021; Sterlin et al., 2021).

Next, the diversity of B-cell response repertoire in B-cell subtypes was assessed to investigate the heterogeneity of B-cell response in COVID-19. Here, sequences of the third complementarity determining region of the antibody heavy chain (CDRH3) were analyzed. Comparison of homogenous scores of the B-cell response repertoire in B-cell subtypes from moderate and severe patients revealed a significantly higher homogenous score in plasmablasts and plasma cells in the moderate group over the severe group (**Figure 2B**). We failed to detect proliferating clonotype sequences in our dataset potentially due to limited B-cell numbers. Nevertheless, these suggest that increased heterogeneous B-cell responses with more diverse B-cell response repertoires occur in severe patients than in moderate cases during plasma cell differentiation (**Figure 2B**). The increased heterogeneous B-cell response response in severe COVID-19 appears to be specific to plasmablasts and plasma cells since the homogenous B-cell response scores were not significantly different in naïve and memory cells between moderate and severe patients. In addition, severe patients developed significantly higher level of anti-spike RBD IgG than those in moderate group and the average homogeneity score of B-cell response sequences from the patients inversely correlated with the spike-specific antibody titers (**Figures 2B, C**), suggesting more diverse B-cell response repertoires supporting enhanced IgG antibody responses. When we examined anti-spike RBD IgA and SARS-CoV-2 neutralizing activity in plasma from the patients, there was no significant difference between moderate and severe patients (**Supplementary Figure 6**). The average homogeneity score of B-cell response sequences from the patients negatively correlated, but without statistical significance, with the spike-specific IgA or neutralizing antibody titers. Therefore, the increased heterogeneous B-cell response response in severe COVID-19 seems to be more specifically associated with higher level of antigen-specific IgG, but not with neutralizing activity, in our small cohort.

### Enhanced Extrafollicular B-Cell Response With Aberrant Plasma Cell Differentiation in Severe COVID-19

**Figure 3:**
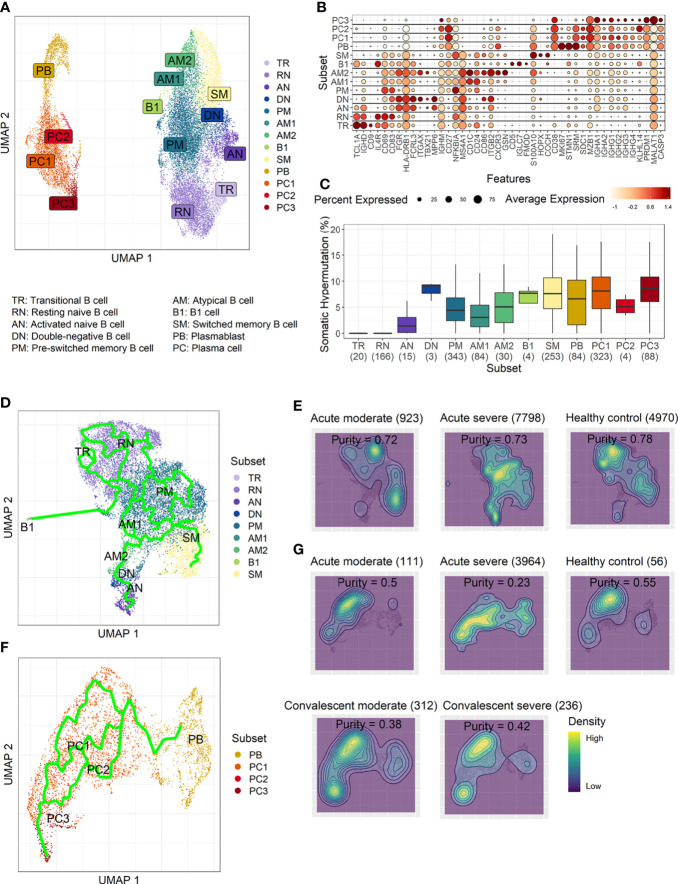
Characterization of B-cell subsets in detail using transcriptional signatures and somatic hypermutation rate. **(A)** Uniform Manifold Approximation, and Projection (UMAP) for clustered B-cell subsets. **(B)** Dot plot for the most significant DEGs of each subset. Diameter presents the percentage of cells expressing the DEG and with color for expression level. **(C)** Boxplots for a percentage of somatic hypermutation rate of IGH of the indicated subset. The number of BCR sequences used for one boxplot is stated within the blanket. **(D)** Trajectory UMAP for subsets of naïve and memory B cells with lines of potential differentiation lineages. **(E)** Cell density on trajectory UMAP of naïve and memory B-cell subsets. The number of cells and expression purity is displayed for the indicated patient group. **(F)** Trajectory UMAP for plasmablasts and plasma cell subsets. **(G)** Cellular densities on the UMAP of plasmablasts and plasma cell subsets.

To develop a deeper understanding of the nature of B-cell responses in COVID-19, we further divided the B-cell population into more detailed subclusters using their transcriptional signatures (Jenks et al., 2018; Woodruff et al., 2020) and SHM rate obtained from scRNA datasets (**Figures 3A–C**). Previous high-dimensional flow cytometry data indicated that severe COVID-19 patients displayed hallmarks of EF B-cell activation and shared B-cell repertoire features in autoimmune settings (Woodruff et al., 2020). Therefore, our study primarily focused on B-cell subsets representing EF responses. Here, 13 B-cell subclusters, including 3 subsets of plasma cells and 5 subsets of memory B cells, were identified by cluster analysis on UMAP (**Figure 3A**) (Becht et al., 2019). IGHD-expressing naïve B cells were subdivided into TR B cells, resting naïve (RN) B cells, and activated naïve (AN) B cells according to the differential expression of the specific signature gene set (**Figure 3B**). The AN B cells were IGHD-positive clusters with comparatively higher SHM rates (**Figure 3C**) than other naïve B cells and expressed distinct genes, such as FGR and MPP6. During the EF response, AN B cells differentiate into effector cells lacking naïve (IgD) and memory (CD27) markers or DN B cells (Woodruff et al., 2020). DN B cells showed a higher SHM rate than AN B cells and expressed ITGAX (CD11C) and CD86 (**Figures 3B, C**). A previous study also reported that the human EF effector B-cell responses are mediated by the expansion of a unique population of CD11c^+^ AN B cells (Jenks et al., 2018). Two clusters of atypical memory (AM1 and AM2) B cells were identified within the memory B-cell compartment, which overexpressed CD1C. Interestingly, AM2 B cells shared several transcriptional signatures, including FGR and HLA-DRB1, with EF B-cell subsets, including AN and DN cells. Both AM2 and DN B cells express ITGAX, TBX21, CD86, and ITGB2, but AM2 uniquely expressed CXCR3 and GSN (gelsolin).

Among three plasma cell subtypes, plasma cell 2 (PC2) possessed features unique from the other plasma cells, such as overexpression of KLHL14 and a moderately lower SHM rate (**Figures 3B, C**). Recently, it was shown that KLHL14 reduces overall B-cell response levels through promoting ubiquitylation of the B-cell response subunits, which decreases the stability of immature B-cell response glycoforms in the endoplasmic reticulum (Choi et al., 2020). Consistently, PC1 presented standard class-switching features (IGHA2, IGHG1, and IGHG2), whereas PC2 primarily expressed IGHM with a lower SHM rate. These differences suggest a delayed isotype switching process and exclusion from the GC reaction. Conversely, PC3 overexpressed PRDM1 and MALAT1, which are genes relating to plasma cell maturation and apoptosis, respectively (Li et al., 2018), suggesting that these cells were in the end stage of plasma cell differentiation (**Figures 3B, C**).

Trajectory analysis for potential lineage association revealed that AN, DN, and AM2 B-cell subsets were closely linked sequentially (**Figure 3D**), highlighting the pathway of EF B-cell response as “AN → DN → AM2”. In addition, cellular density overlaid on the trajectory UMAP showed differential B-cell responses depending on COVID-19 severity (**Figure 3E**). Indeed, severe patients presented a higher proportion of preswitched memory (PM) B cells and the lineages (AN, DN, and AM2) of EF B-cell response during the acute stage. In contrast, moderate cases illustrated denser B-cell subsets of RN and switched memory (SM) cells with few PM and EF B cells. Expression purity was also calculated by entropy-based statistics to determine the variation between transcriptional profiles in specific subtypes among the patients, as well as within the individuals’ cellular populations (Liu et al., 2020). Moderate (0.72) and severe (0.73) patients in the acute stage exhibited similar expression concordance as that in the healthy controls (0.78) (**Figure 3E**).

**Figure 4:**
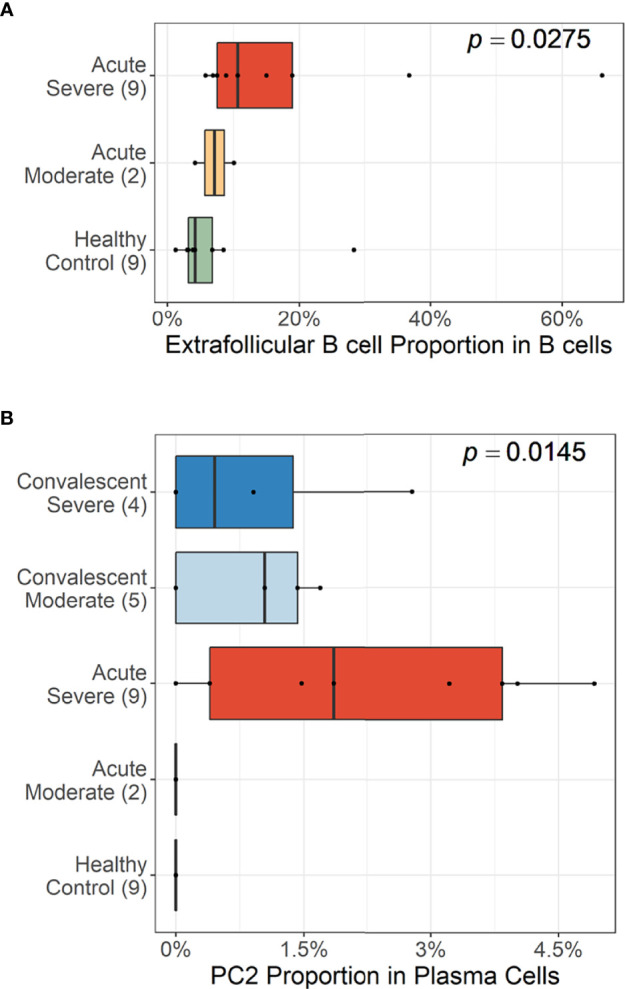
Cellular proportions of extrafollicular (EF) B cells (AN, DN, and AM2) in total B-cell population **(A)** and plasma cell 2 (PC2) among plasma cells **(B)**. *p*-values were calculated by the Kruskall–Wallis test. The number of patients in each group is presented within the blanket.

Trajectory analysis performed on plasma cells clearly illustrated a differentiation pathway from plasmablasts to PC3, either through PC1 or PC2 (**Figure 3F**). Interestingly, a clear variation of cellular density distribution existed between the moderate and severe patients during the acute phase (**Figure 3G**, upper panels). Severe cases presented denser plasmablasts and slightly biased cellular density toward the PC2 subset with widely spreading over the plasma cell population during the acute stage. Conversely, the patients with moderate cases showed more convergent patterns toward PC1. Moreover, the calculated expression purity also confirmed higher variation in gene expression in the severe group (0.23), yet a relatively lower expression purity value when compared to the moderate patients (0.5). The observed difference in cellular distribution between the two groups during the acute phase became marginal during the convalescent stage, where both groups showed PC1-biased cellular densities, as well as higher PC3 at the terminal stage (**Figure 3G**). The relative proportion of B-cell population associated EF B-cell response (AN, DN, and AM2), as well as the aberrant PC2 subset, was significantly higher in severe patients, not in moderate group, during the acute phase of infection when compared to the healthy control (**Figure 4**). Moreover, while the proportion of putative EF B-cell subsets (AN and AM B cells) was not statistically significant (*p* = 0.2), it was noticeably higher in deceased patients compared to those who recovered in ICU (**Supplementary Figure 7**).

### Inflammatory Features of Extrafollicular B-Cell Response and Aberrant Plasma Cell, PC2

**Figure 5:**
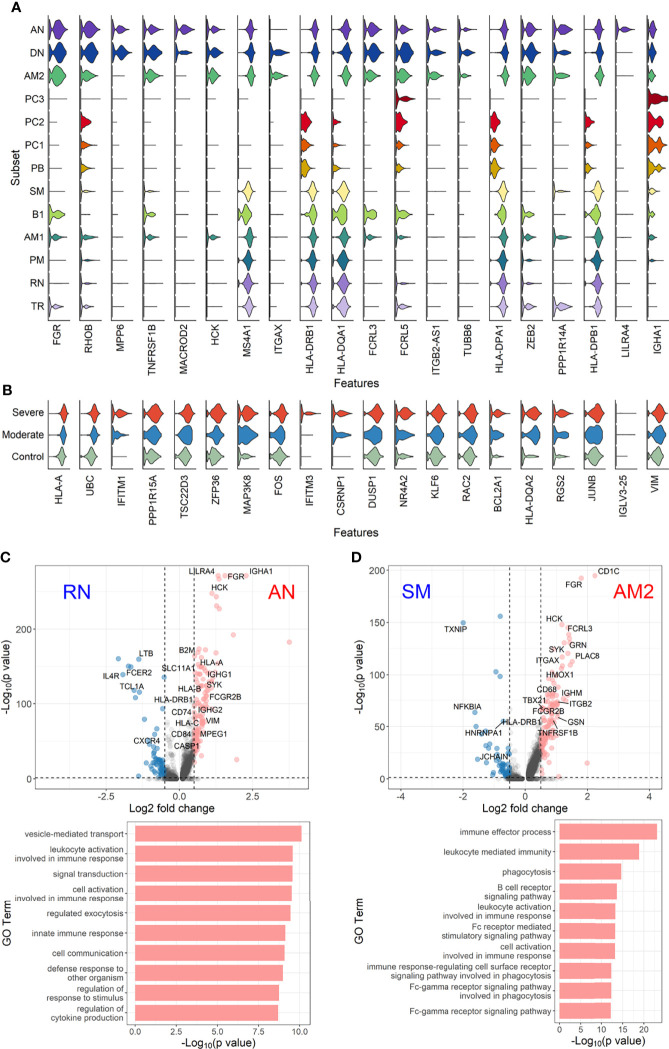
Characterization of EF B-cell subsets. **(A)** Gene expression violin plots of the 20 most significant DEGs in three EF B-cell subsets (AN, DN, and AM2) in comparison with other B-cell subsets. **(B)** Violin plots of top 20 DEGs in the severe group across three EF B-cell subsets. Volcano plot and bar plot presenting significant DEGs and top 10 significant GO terms when compared between AN and RN B-cell subsets **(C)**, or between AM2 and SM B-cell subsets **(D)**.

To further define the transcriptional features of EF B-cell subsets (AN, DN, and AM2) and their functional potential associated with COVID-19 severity, we analyzed DEGs in the EF B-cell population by comparing them with those in other B-cell subsets (**Figure 5**). Interestingly, Src family kinase–related genes, including HCK, FGR, FCRL3, and FCRL5, were generally more overexpressed in EF B-cell subsets than in other B-cell subsets. In addition, ITGB2-AS1 and TUBB6 were uniquely expressed in the three EF B-cell subsets, which suggests novel hallmark signatures related to EF B-cell responses. Expression of IGHA1 was relatively higher in EF B-cell subsets than in other memory B-cell populations, except plasma cell subtypes (**Figure 5A**). Analysis of the transcriptional markers associated with disease severity in the three EF B-cell subsets revealed that the top 20 DEGs enriched in the severe group expressed a pattern of gradual increment, which transitioned sequentially from the healthy control to the moderate group and finally into the severe group (**Figure 5B**). GO term analysis of the top 20 DEGs matched to the antigen processing function as indicated by significant increase of HLA-A and HLA-DQA2 in the severe group, which correlates to a previous association with COVID-19 severity (Shkurnikov et al., 2021). Similarly, other genes involved in type I interferon signaling (IFITM1 and IFITM3) and cellular activation (UBC, FOS, and DUSP1) were increased in a severity-dependent manner (**Figure 5B**), suggesting an enhanced EF B-cell activity associated with an overt inflammatory condition in severe COVID-19.

**Figure 6:**
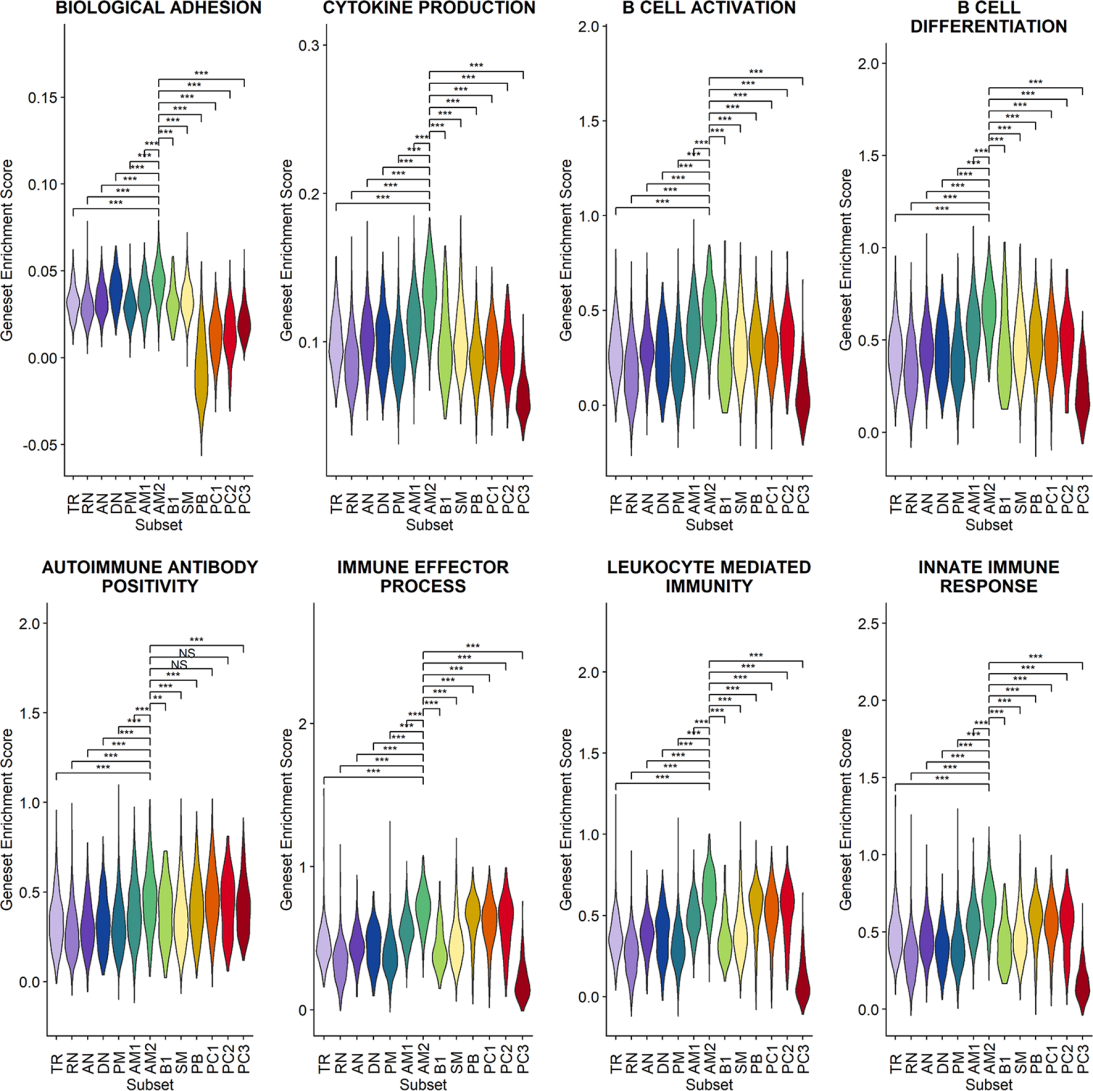
Characterization of AM2 B-cell subsets by gene set enrichment analysis for inflammatory functions in comparison with other B-cell subsets. Statistical significance was calculated by Wilcoxon rank sum test: ***p < 0.0001; **p < 0.001; *p < 0.01. NS, not significant.

Since a significant elevation of transcriptional features involved in inflammation and cellular activation in EF B-cell subsets was detected, we further confirmed DEGs and their functional characteristics between EF B cells and their counterpart subsets (**Figures 5C, D**). Compared to RN, the AN subsets expressed relatively higher levels of the class-switched immunoglobulin isotype (IGHA1) and B-cell signaling regulators, such as FGR, HCK, and CD74 (Gil-Yarom et al., 2017). Following GO analysis, the DEG gene set from AN was also highly associated with the immune response and cellular activation functions (**Figure 5C**), whereas RN contained gene sets involved in cell recognition and metabolic processes (**Supplementary Figure 8**). Among memory B-cell subtypes, DEGs in AM2 and SM were compared to define the transcriptional features of atypical memory B cell associated with the EF B-cell response. Indeed, CD1C and HLA-DRB1 were significantly higher in AM2 than in the SM subset (**Figure 5D**). Furthermore, the immune effector process and B-cell signaling were upregulated in the primary functional DEGs categories of AM2, whereas a higher level of GC-related genes, including TXNIP and JCHAIN, were expressed in SM (**Figure 5D**). Likewise, were the DEG gene set terms of SM subset involved in ordinary cellular activities such as metabolic process, regulation of respiratory burst, and lymphocyte differentiation (**Supplementary Figure 8**). These results indicate that the AM2 subset is potentially one of the primary effectors associated with the acute inflammatory process resulting from the acute EF B-cell response in severe COVID-19 cases. Although AM2 shared several markers with the AN and DN B EF B-cell subsets, the EF response subset can be distinguished by an increased expression of the specific transcriptional signatures, such as GSN (**Supplementary Figure 9**). The inflammatory functions of AM2 were further characterized by calculating and comparing the gene set enrichment score of immune-related terms among 13 classified B-cell subsets. Here, AM2 cells presented significantly higher transcriptional profiles for biological adhesion, cytokine production, B-cell activation, leukocyte-mediated immunity, and innate immune response (**Figure 6**). Meanwhile, a functional category of autoimmune antibody positivity was relatively higher in AM2 compared to other B-cell subsets, except in plasma cells, PC1 and PC2. Analysis of the gene set enrichment score specifically enhanced in the aberrant plasma cell population (PC2) displayed three inflammatory signature categories that were significantly higher than in the other plasma cell subsets (complement activation, immunoglobulin complex, and phagocytosis recognition) (**Supplementary Figure 10**). This suggests that the PC2 inflammatory subset could be one of the primary cells associated with severe disease progress in COVID-19.

## Discussion

Despite the massive global endeavors to identify the pathogenic mechanisms of COVID-19, the primary culprits involved in the severe pneumonic progression leading to ARDS and lethal outcomes remain elusive. Yet, a dysregulated macrophage response and a macrophage activation syndrome have been identified as a potential cause of severe COVID-19 progression (Merad and Martin, 2020). The presence of substantial evidence has supported the notion that aberrant immune responses to human coronaviruses are typified by the dysregulation of the innate response, including type I and III interferons, alongside an aberrant adaptive immune response against the invading pathogen (Wong and Perlman, 2022). Recently, our group reported that enhanced eosinophil-mediated inflammation and subsequent pulmonary pathogenesis *via* enhanced T_H_2-biased immune responses have been associated with increased formation of immune complexes, and membrane attack complexes in airways and vasculature of inflamed lungs, especially in critical COVID-19 (Kim et al., 2021). In addition, previous seminal studies have reported the potential pathogenic role of dysregulated humoral immune responses, including overt EF B-cell activation (Woodruff et al., 2020) and autoantibodies (Bastard et al., 2020; Wang et al., 2021), correlated with severe COVID-19. Nevertheless, the mechanistic pathway to the aberrant humoral response involved in the pathogenic progression of severe COVID-19 is largely unclear. Here, we investigated phenotypical characteristics of B-cell differentiation in detail using scRNA sequencing analysis on circulating B cells to drive a deeper understanding of the pathogenic nature of B-cell responses in association with COVID-19 morbidity. Through this study, we can identify an EF B-cell response differentiation pathway (AN → DN → AM2), which is associated with COVID-19 progression and severity. Additionally, we identified an aberrant plasma cell subset, PC2, response as a potential driver of aggravating humoral response.

Our results first confirmed that there is an enhanced plasma cell response in more severe disease cases (**Figure 1**). Furthermore, there is a preferential isotype-switching toward IgA response in plasmablasts and plasma cells from COVID-19 patients (**Figure 2A**), as reported previously (Sosa-Hernandez et al., 2020; Bernardes et al., 2020; Galson et al., 2020; Zeng et al., 2021; Sterlin et al., 2021). Interestingly, homology analysis on CDRH3 sequences of immunoglobulin transcripts revealed there are more heterogeneous plasma cell responses with diverse B-cell response repertoires in severe patients than in moderate group. In addition, severe patients developed significantly higher level of anti-spike RBD IgG responses than those in moderate group and the average homogeneity score of B-cell response sequences from the patients inversely correlated with the spike-specific antibody titers. Findings in the severe group of enhanced plasma B-cell responses with more B-cell response diversity, together with higher levels of specific antibodies to the invading virus (**Figures 2B, C**), suggest more rapid and heterogeneous plasma cell responses supporting more robust production of specific antibodies. This is probably due to extensive EF B-cell activation (Woodruff et al., 2020), which could be a hallmark of severe COVID-19. Furthermore, we have also provided evidence of a correlation between COVID-19 severity and EF B-cell response, as well as their differentiation trajectory from AN B cells toward AM2 B cells *via* DN population (**Figures 3**, **4**). In addition, FGR was identified as a common transcriptional signature of these three EF B-cell subsets and GSN as a novel and specific identifier for the AM2 population (**Figure 3B**). FGR encodes an Src kinase family member, feline Gardner–Rasheed sarcoma viral oncogene homolog, which, together with another Src-family kinase, HCK, was among the most strongly upregulated transcripts in the specific memory B-cell subsets, FcR-like (FCRL)–positive AM B-cell subset (Liu et al., 2015; Li et al., 2020). The FCRL family members are immunoreceptor tyrosine-based inhibitory motif– and/or immunoreceptor tyrosine-based activation motif–bearing cell surface receptors, are often most highly expressed by AM B cells, and phosphorylated by FGR and HCK. Phosphorylation promotes the modulation of B-cell response signaling upon the binding of an antigen or another stimulating ligand (Ehrhardt et al., 2005; Li et al., 2013). Interestingly, all three EF B-cell subsets identified in our study expressed relatively high levels of FCRL3 and FCRL5, as well as FGR and HCK (**Figures 3B**, **5A**). FCRL3 expression is associated with autoimmune and lymphoproliferative disorders, implying a role in promoting B-cell pathogenesis (Li et al., 2013). A higher frequency of FCRL5^+^ AM B cells was also observed in chronic infections such as malaria and hepatitis C in addition to common variable immunodeficiency and autoimmune conditions, including rheumatoid arthritis and SLE (Li et al., 2020). Therefore, the commonly shared high expression of FCRL3, FCRL5, FGR, and HCK kinases observed in the EF B-cell lineages may represent the underlying mechanisms of AM2 differentiation and their activation, further to their potential involvement in COVID-19 progression by dysregulated humoral effector function. Moreover, the AM2 subset presented significantly higher transcriptional profiles for biological adhesion, cytokine production, B-cell activation, leukocyte-mediated immunity, and innate immune response when compared to other B-cell subsets (**Figure 6**). Although this subset showed a relatively lower SHM rate when compared to SM and plasma cells (**Figure 3C**). Such observations support their potential as an inflammatory driver, rather than a protective producer of high-affinity antibodies. It is also notable that many of the transcriptional signatures, including RHOB, MPP6, TNFRSF1B, ITGB2, and ZEB2 elevated in AM2 from COVID-19 patients have also been detected as transcriptional markers of AM B cells (**Figure 5**). Moreover, they express remarkably similar transcriptional profiles in malaria, HIV, and autoimmune diseases (Holla et al., 2021; Sutton et al., 2021). Additionally, higher expression of GSN was identified in the AM2 subset, suggesting as a specific marker of AM2. It is an actin filament–binding protein regulating the architecture and motility of cells (Burtnick et al., 1997). While previous studies reported the role of GSN in anti-viral immunity (Irving et al., 2012) and its overexpression in activated B cells (Sutton et al., 2021), its specific function in AM B-cell activation and differentiation remains to be elucidated.

A further finding relates to a specific and temporal surge of PC2 subset in severe COVID-19 during the acute phase of infection (**Figures 3G**, **4B**). This subset presented a relatively lower SHM rate in comparison with other plasma cell subsets. Moreover, it expressed mainly IgM antibody transcripts, which suggests that they were short-lived plasma cells derived from an active EF B-cell compartment during the acute phase of COVID-19. Similar IgM-expressing B cells with lower SHM rates were identified in COVID-19 patients in a previous study (Woodruff et al., 2020). Moreover, this B-cell subset presented the highest enriched gene set scores for phagocytic activity, complement activation, and immune complex response when compared to the other B-cell subsets (**Supplementary Figure 10**). These enhanced scores suggest a potential contribution in pathogenic humoral responses observed in severe COVID-19 during early stages (Kim et al., 2021). While we could not define whether the differentiation pathway of PC2 cells is linked to an EF B-cell response pathway (AN → DN → AM2), it is plausible that both the AM2 and PC2 cells seem to function as pathogenic B cells. This potentially occurs through multiple mechanisms, including the generation of inflammatory mediators, production of autoreactive antibodies, and immune complex formation during the acute phase of COVID-19 (Woodruff et al., 2020; Kim et al., 2021; Wang et al., 2021), wherein the GC formation is suppressed (Kaneko et al., 2020).

The potential limitation associated with this study is the use of the B-cell response dataset only from convalescent patients. Thus, the B-cell response sequence changes from the corresponding sample during the acute phase could not be assessed. CD38^+^ plasma B cells were initially sorted and analyzed to focus on the characterization of the activation-associated transcriptional signatures in plasma cells during COVID-19 progression. Nevertheless, any potential sorting bias could be compensated for by extensive validation of transcriptional markers of B cells, exclusion of datasets contaminated by other leukocytes, and the integration with the public B-cell dataset. Moreover, we observed a relatively conserved cellular proportion of B-cell subtypes from the acute and convalescent stage of COVID-19 among the combined data (**Figure 1B**). Owing to the lack of detailed information alongside the use of various sources of data, the clinical and ethical differences were not fully considered in the research. The number of samples used in this study was limited to obtain a statistical significance in several of the performed analyses, thus further research with a larger cohort is required for validation.

## Data Availability Statement

The datasets for this article are not publicly available due to concerns regarding participant/patient anonymity. Requests to access the datasets should be directed to the corresponding author.

## Ethics Statement

The studies involving human participants were reviewed and approved by the Institutional Review Board of National Medical Center (H-2004-116-011) and Seoul National University Hospital (IRB No. C-1509-103-705). The patients/participants provided their written informed consent to participate in this study. Written informed consent was obtained from the individual(s) for the publication of any potentially identifiable images or data included in this article.

## Author Contributions

N-HC and BC conceptualized this study. N-HC, BC TL, and YK designed the experiment and wrote the manuscript. TL, YK, HK, N-YH, and SL performed the experiments and data analyses. BC oversaw the collection and summarizing of the clinical data. All authors contributed to the article and approved the submitted version.

## Funding

This research was supported by a grant from the Korea National Institute of Health: the Korea Disease Control and Prevention Agency (HD20A0533), a grant from the National Research Foundation of Korea (grant no. 2021M3A9I2080490), and the 2022 Joint Research Project of Institutes of Science and Technology (to N-HC).

## Conflict of Interest

Authors: TL and SL, are employed by Geninus Inc.

The remaining authors declare that the research was conducted in the absence of any commercial or financial relationships that could be construed as a potential conflict of interest.

## Publisher’s Note

All claims expressed in this article are solely those of the authors and do not necessarily represent those of their affiliated organizations, or those of the publisher, the editors and the reviewers. Any product that may be evaluated in this article, or claim that may be made by its manufacturer, is not guaranteed or endorsed by the publisher.

## Supplementary Material

Supplementary Figure 1 Integration and filtering of B cell population from scRNA datasets. **(A)** UMAP analysis for selecting pure B cell population and removal of other leukocytes population contaminated in the integrated datasets. Cellular distribution of original scRNA sequencing dataset (red dots) from acute patients **(B)**, convalescent patients (current study), and healthy control dataset **(C)** imposed on the integrated dataset (gray). **(E)** Validation of B cell-specific transcriptional makers (CD19, CD22, CD27, and CD38) and contaminated cellular populations indicated by myeloid cell (CD14)-, T cell (CD3D)-, NK cell (NCR1)-, and platelet (PPBP)-specific transcripts in the integrated datasets.

Supplementary Figure 2 UMAP analysis of selected B cell population from convalescent patients **(A)** and acute patients **(B)**. Annotated cell types are indicated with their numbers and percentage among whole B cell population. Cellular distribution of individual sample from convalescent patients **(C)** and acute patients **(D)** imposed on integrated UMAP.

Supplementary Figure 3 Cellular distribution of B cells from individual patient on trajectory UMAP of naïve and memory B cells (see ).

Supplementary Figure 4 Cellular distribution of B cells from individual patient on trajectory UMAP of plasmablasts and plasma cells (see ).

Supplementary Figure 5 Boxplots for cellular proportions of B cells **(A)** and plasma cells **(B)** in PBMCs. Statistical significance was calculated by Wilcoxon rank sum test: ***, *p*<0.0001. NS, not significant.

Supplementary Figure 6 Anti-spike RBD IgA response and neutralizing activity in plasma from COVID-19 patients. **(A)** Violin plot presenting anti–SARS-CoV-2 spike RBD-specific titers for IgA (left panel) and correlation of anti-RBD-specific IgA titers with average homogeneity score of COVID-19 patients (right panel). **(B)** Violin plot presenting anti–SARS-CoV-2 FRNT_50_ (left panel) and correlation of neutralizing titers with average homogeneity score of COVID-19 patients (right panel). Statistical significance was calculated by Pearson correlation.

Supplementary Figure 7 Difference in tentative EF B cell subsets (AN and AM) proportion among naïve and memory B cell population between survived and deceased patients after admission in ICU. Tentative EF B cells were identified from the B cell dataset reported previously (Bost et al., 2021). Plasma cells were barely detected in this dataset due to poor data quality.

Supplementary Figure 8 Bar plots of significant GO terms significantly upregulated in the indicated B cell subsets (RN and SM) when compared to AN and AM2, respectively (see ).

Supplementary Figure 9 Heatmap of representative DEGs showing the statistically significant difference among three EF B cell subsets.

Supplementary Figure 10 Computed hallmark gene set activity scores of indicated categories among B cell subsets. Statistical significances between AM2 and other B cell subsets were calculated by Wilcoxon rank sum test: ***, *p*<0.0001; **, *p*<0.001; *, *p*<0.01.
