# Corrigendum: Acute surge of atypical memory and plasma B-cell subsets driven by an extrafollicular response in severe COVID-19

**DOI:** 10.3389/fcimb.2023.1178630

**Published:** 2023-03-22

**Authors:** Taeseob Lee, Yuri Kim, Hyun Je Kim, Na-Young Ha, Siyoung Lee, BumSik Chin, Nam-Hyuk Cho

**Affiliations:** ^1^ Department of Digital Health, Samsung Advanced Institute for Health Sciences & Technology, Sungkyunkwan University, Seoul, Republic of Korea; ^2^ Discovery Department, Biomarker Laboratory, Geninus Inc., Seoul, Republic of Korea; ^3^ Institute of Endemic Diseases, Medical Research Center, Seoul National University, Seoul, Republic of Korea; ^4^ College of Medicine, Genome Medicine Institute, Seoul National University, Seoul, Republic of Korea; ^5^ School of Medicine, Biomedical Research Institute, Chungnam National University, Daejeon, Republic of Korea; ^6^ Department of Internal Medicine, National Medical Center, Seoul, Republic of Korea; ^7^ Department of Microbiology and Immunology, College of Medicine, Seoul National University, Seoul, Republic of Korea; ^8^ Department of Biomedical Sciences, College of Medicine, Seoul National University, Seoul, Republic of Korea; ^9^ Bundang Hospital, Seoul National University, Seongnam, Republic of Korea; ^10^ Wide River Institute of Immunology, Seoul National University, Hongcheon, Republic of Korea

**Keywords:** B cells, extrafollicular response, COVID-19, antibody response, plasma cell

 

