# Supplementary figures and images for: Acute Surge of Atypical Memory and Plasma B-Cell Subsets Driven by an Extrafollicular Response in Severe COVID-19
Source: Front Cell Infect Microbiol. 2022 Jul 8;12:909218. doi: 10.3389/fcimb.2022.909218 (PMC9309264; doi:10.3389/fcimb.2022.909218)

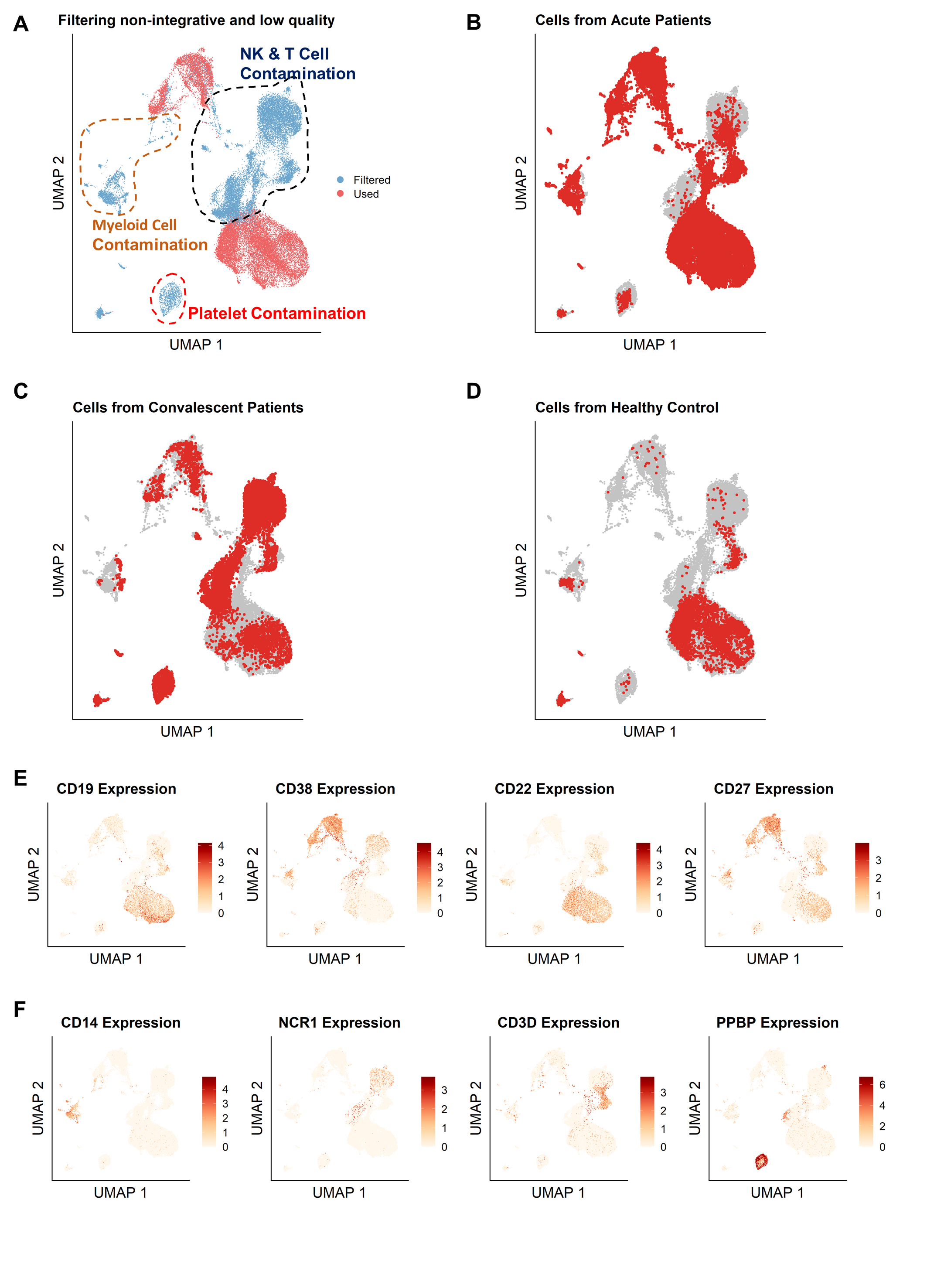

Supplement: Supplementary Figure 1 — Integration and filtering of B cell population from scRNA datasets. (A) UMAP analysis for selecting pure B cell population and removal of other leukocytes population contaminated in the integrated datasets. Cellular distribution of original scRNA sequencing dataset (red dots) from acute patients (B), convalescent patients (current study), and healthy control dataset (C) imposed on the integrated dataset (gray). (E) Validation of B cell-specific transcriptional makers (CD19, CD22, CD27, and CD38) and contaminated cellular populations indicated by myeloid cell (CD14)-, T cell (CD3D)-, NK cell (NCR1)-, and platelet (PPBP)-specific transcripts in the integrated datasets. [file Image_1.tif]

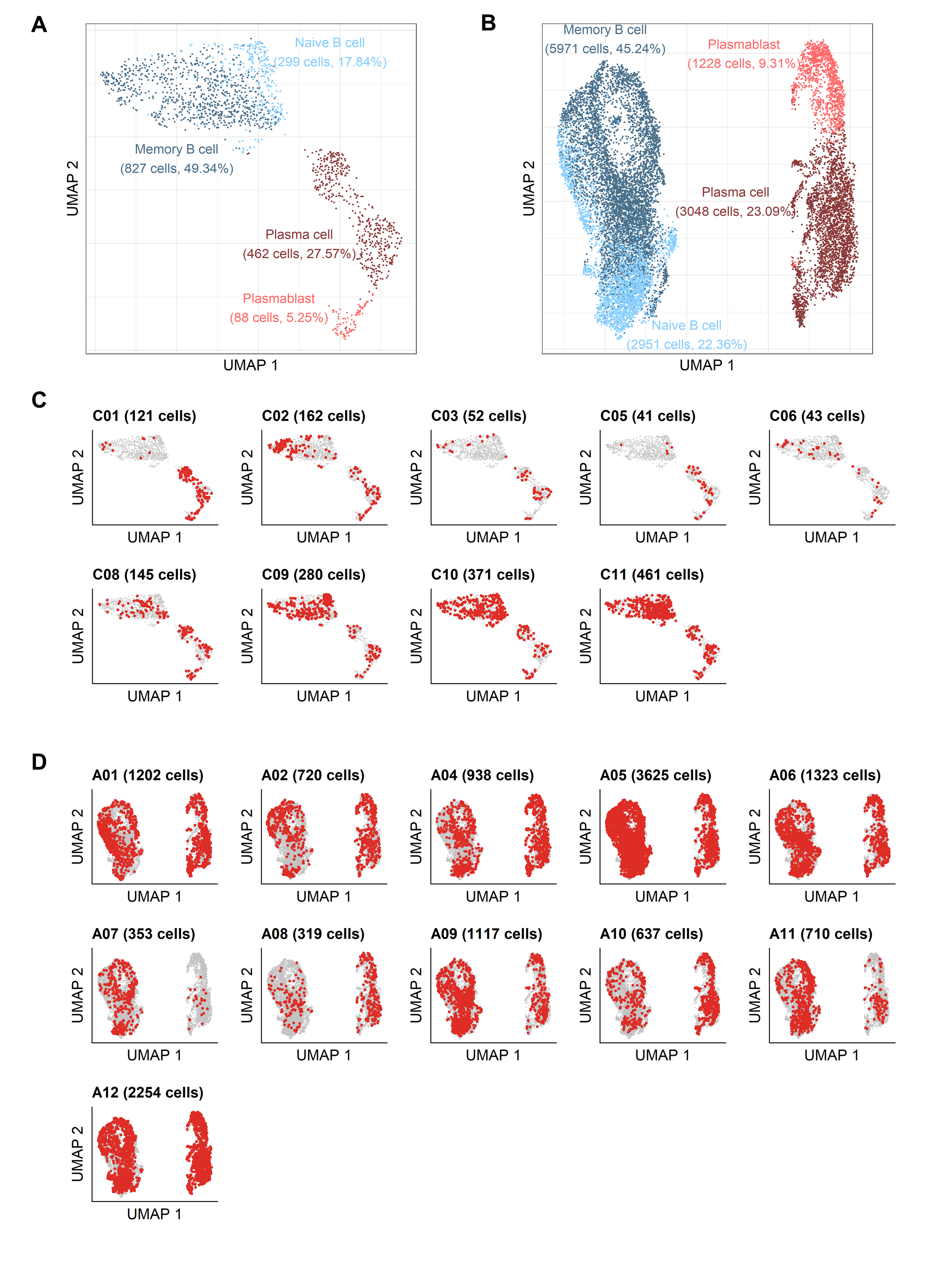

Supplement: Supplementary Figure 2 — UMAP analysis of selected B cell population from convalescent patients (A) and acute patients (B). Annotated cell types are indicated with their numbers and percentage among whole B cell population. Cellular distribution of individual sample from convalescent patients (C) and acute patients (D) imposed on integrated UMAP. [file Image_2.tif]

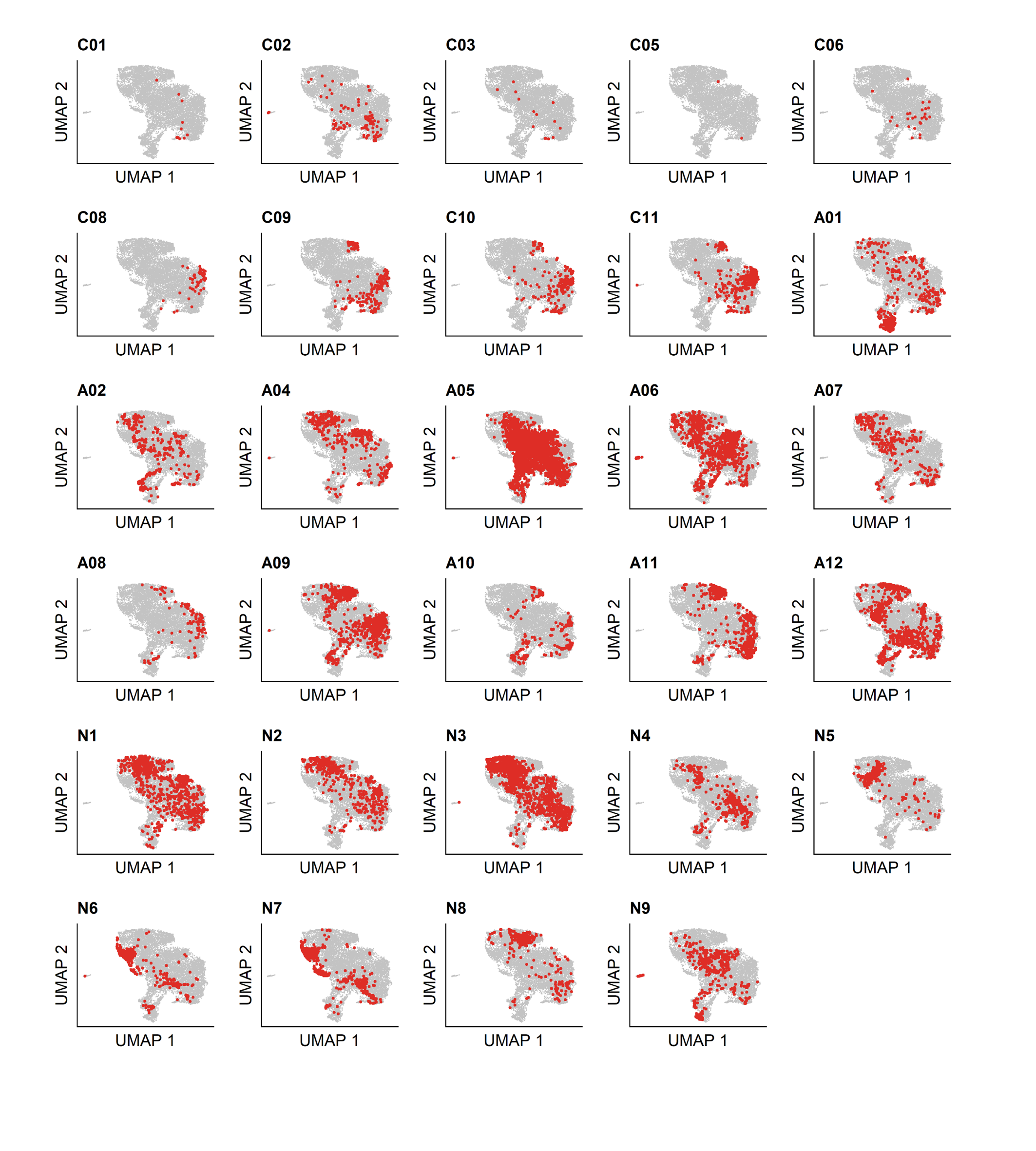

Supplement: Supplementary Figure 3 — Cellular distribution of B cells from individual patient on trajectory UMAP of naïve and memory B cells (see ). [file Image_3.tif]

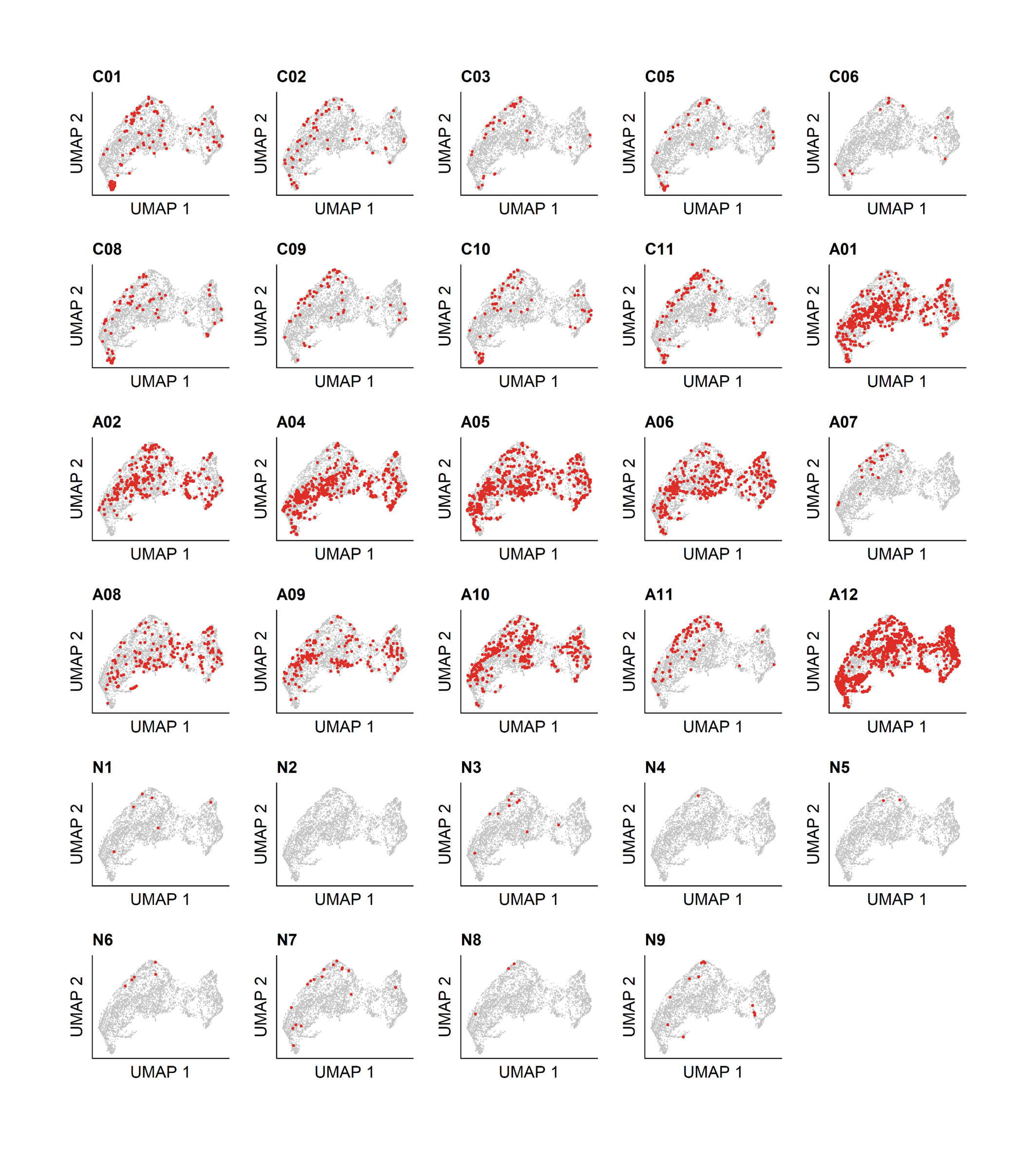

Supplement: Supplementary Figure 4 — Cellular distribution of B cells from individual patient on trajectory UMAP of plasmablasts and plasma cells (see ). [file Image_4.tif]

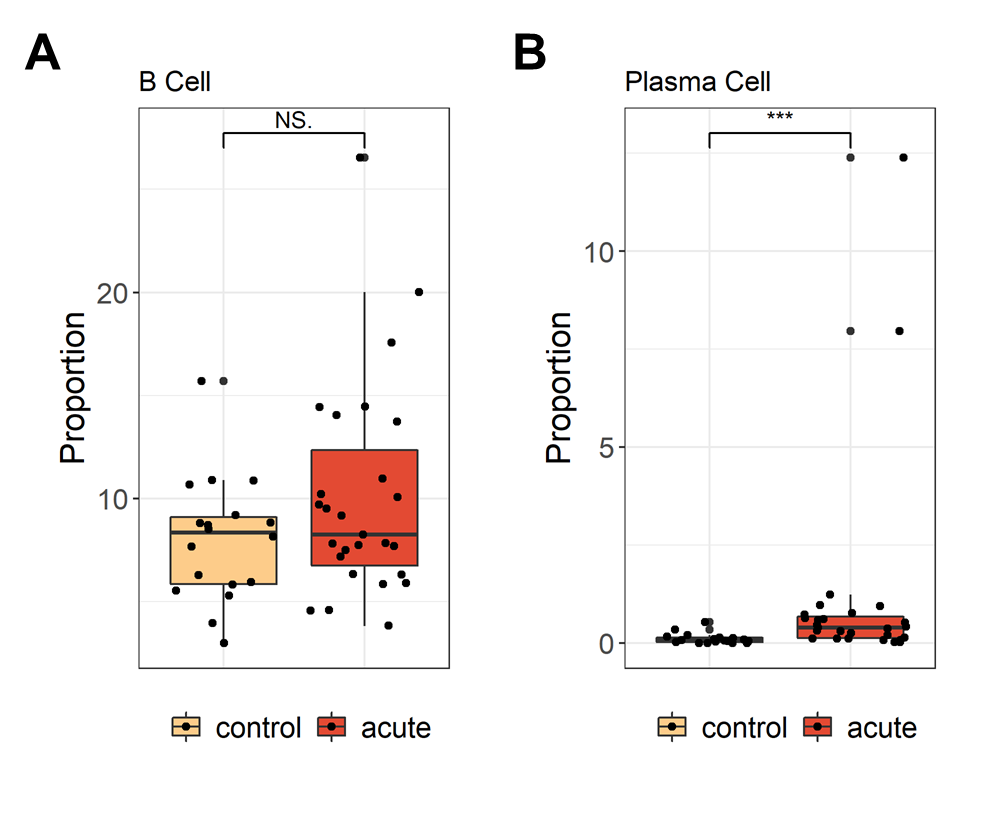

Supplement: Supplementary Figure 5 — Boxplots for cellular proportions of B cells (A) and plasma cells (B) in PBMCs. Statistical significance was calculated by Wilcoxon rank sum test: ***, p<0.0001. NS, not significant. [file Image_5.tif]

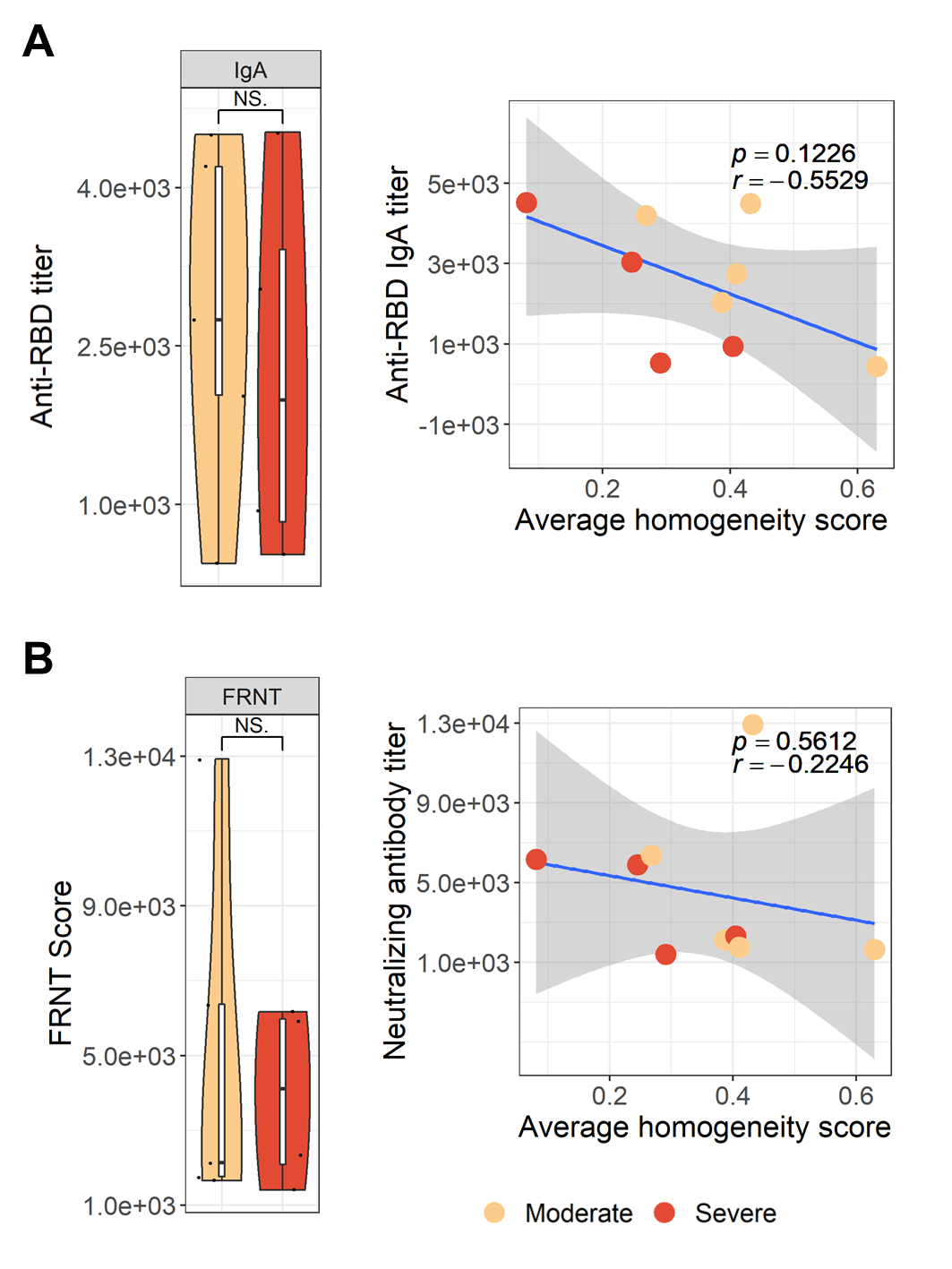

Supplement: Supplementary Figure 6 — Anti-spike RBD IgA response and neutralizing activity in plasma from COVID-19 patients. (A) Violin plot presenting anti–SARS-CoV-2 spike RBD-specific titers for IgA (left panel) and correlation of anti-RBD-specific IgA titers with average homogeneity score of COVID-19 patients (right panel). (B) Violin plot presenting anti–SARS-CoV-2 FRNT50 (left panel) and correlation of neutralizing titers with average homogeneity score of COVID-19 patients (right panel). Statistical significance was calculated by Pearson correlation. [file Image_6.tif]

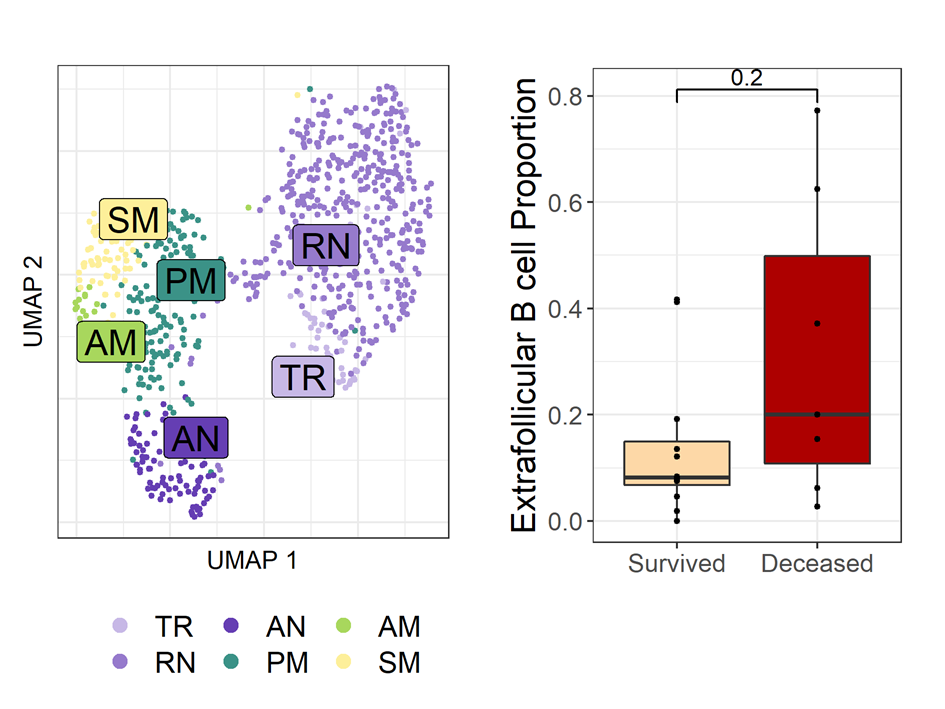

Supplement: Supplementary Figure 7 — Difference in tentative EF B cell subsets (AN and AM) proportion among naïve and memory B cell population between survived and deceased patients after admission in ICU. Tentative EF B cells were identified from the B cell dataset reported previously (Bost et al., 2021). Plasma cells were barely detected in this dataset due to poor data quality. [file Image_7.tif]

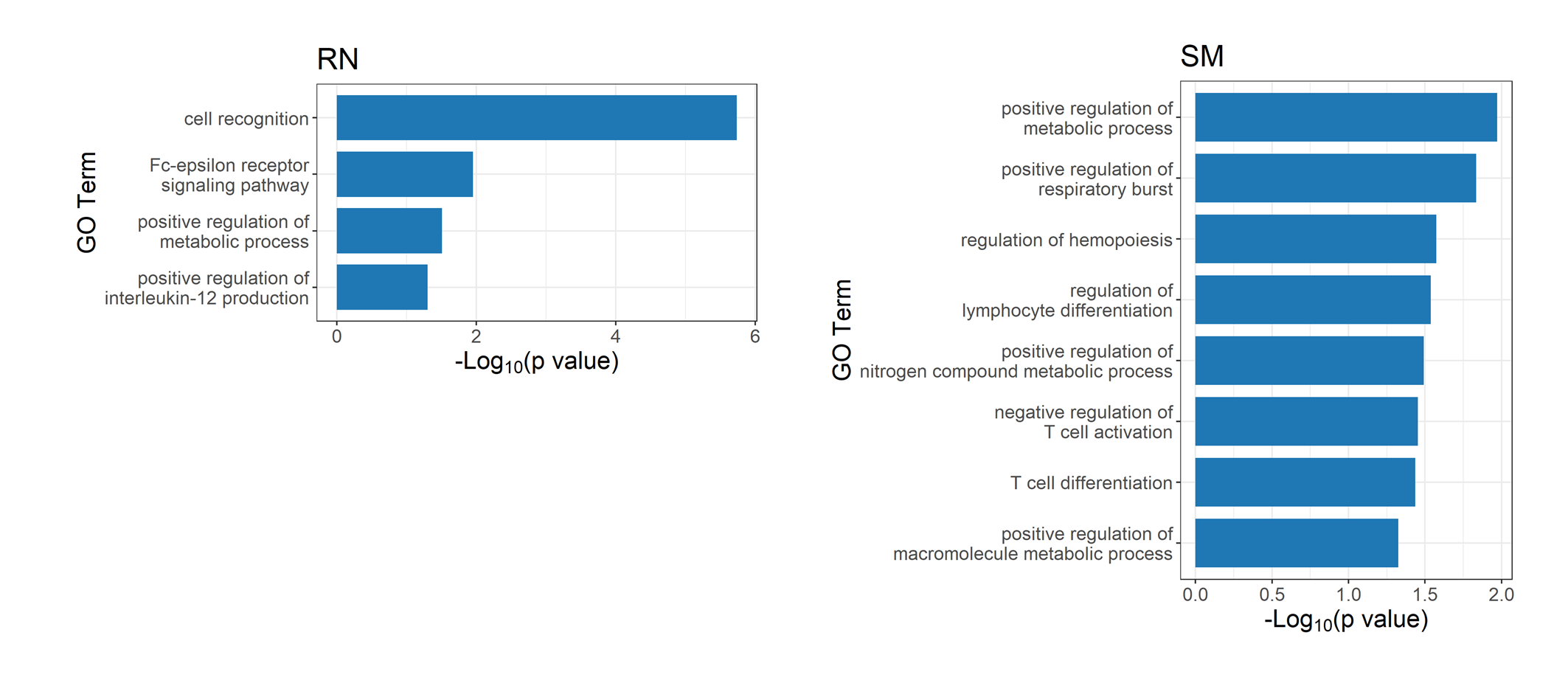

Supplement: Supplementary Figure 8 — Bar plots of significant GO terms significantly upregulated in the indicated B cell subsets (RN and SM) when compared to AN and AM2, respectively (see ). [file Image_8.tif]

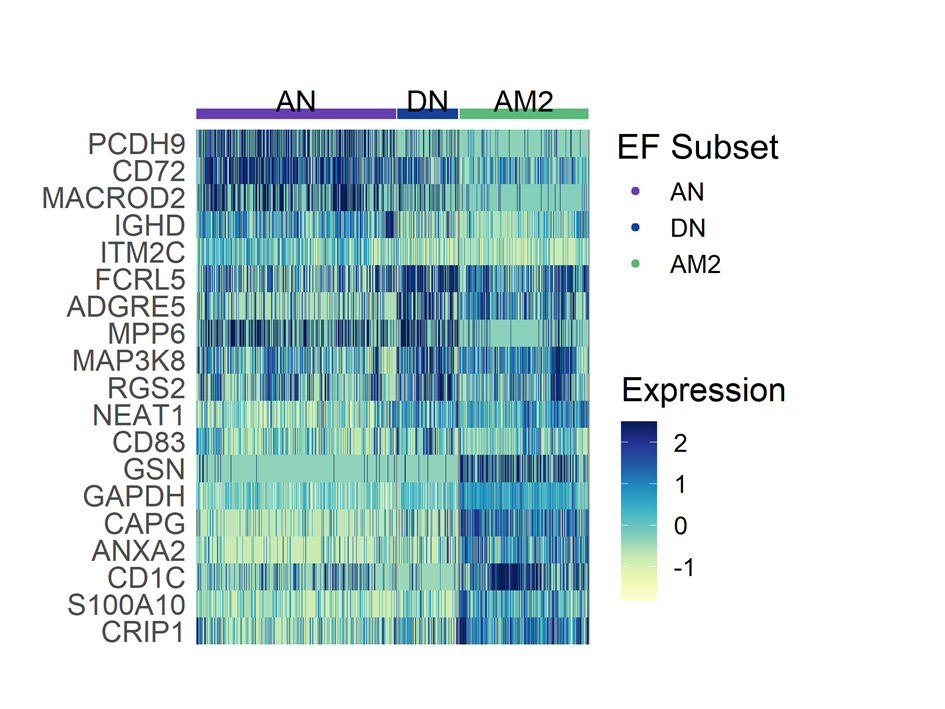

Supplement: Supplementary Figure 9 — Heatmap of representative DEGs showing the statistically significant difference among three EF B cell subsets. [file Image_9.tif]

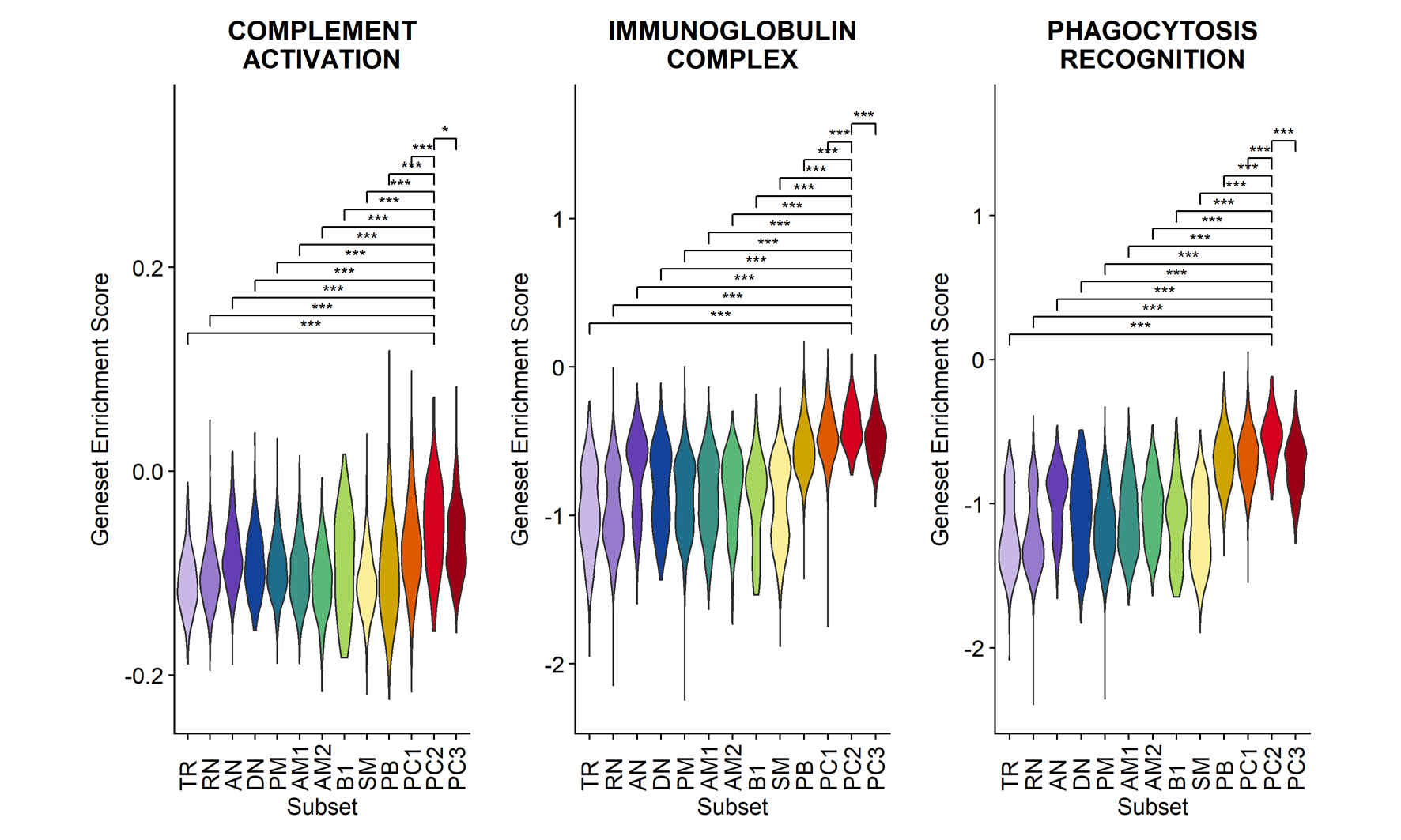

Supplement: Supplementary Figure 10 — Computed hallmark gene set activity scores of indicated categories among B cell subsets. Statistical significances between AM2 and other B cell subsets were calculated by Wilcoxon rank sum test: ***, p<0.0001; **, p<0.001; *, p<0.01. [file Image_10.tif]
